# α-Viniferin and ε-Viniferin Inhibited TGF-β1-Induced Epithelial-Mesenchymal Transition, Migration and Invasion in Lung Cancer Cells through Downregulation of Vimentin Expression

**DOI:** 10.3390/nu14112294

**Published:** 2022-05-30

**Authors:** Wei-Chung Chiou, Cheng Huang, Zi-Jun Lin, Lian-Sheng Hong, Yu-Heng Lai, Jui-Chieh Chen, Hsiu-Chen Huang

**Affiliations:** 1 Laboratory Science in Medicine, Department of Biotechnology, National Yang Ming Chiao Tung University, Taipei 11221, Taiwan; ryanw.chiou@gmail.com (W.-C.C.); chengh@nycu.edu.tw (C.H.); 2 Center for Teacher Education, National Tsing Hua University, Hsinchu 300, Taiwan; maplemouse0526@gmail.com (Z.-J.L.); lshong@mx.nthu.edu.tw (L.-S.H.); 3 Department of Applied Science, Nanda Campus, National Tsing Hua University, Hsinchu 300, Taiwan; 4 Department of Chemistry, Chinese Culture University, Taipei 11114, Taiwan; lyh21@ulive.pccu.edu.tw; 5 Department of Biochemical Science and Technology, National Chiayi University, Chiayi City 60004, Taiwan; jcc@mail.ncyu.edu.tw

**Keywords:** TGF-β1, epithelial-mesenchymal transition, vimentin, α-viniferin, ε-viniferin, nude mice

## Abstract

Resveratrol has well-known anticancer properties; however, its oligomers, including α-viniferin, ε-viniferin, and kobophenol A, have not yet been well investigated. This is the first study examining the anti-epithelial-mesenchymal transition (EMT) effects of α-viniferin and ε-viniferin on A549, NCI-H460, NCI-H520, MCF-7, HOS, and U2OS cells. The results showed that α-viniferin and ε-viniferin significantly inhibited EMT, invasion and migration in TGF-β1- or IL-1β-induced non-small cell lung cancer. α-Viniferin and ε-viniferin also reversed TGF-β1-induced reactive oxygen species (ROS), MMP2, vimentin, Zeb1, Snail, *p*-SMAD2, p-SMAD3, and ABCG2 expression in A549 cells. Furthermore, ε-viniferin was found to significantly inhibit lung metastasis in A549 cell xenograft metastatic mouse models. In view of these findings, α-viniferin and ε-viniferin may play an important role in the prevention of EMT and cancer metastasis in lung cancer.

## 1. Introduction

Lung cancer is highly fatal. The lung cancer five-year survival rate (15%) in the world is lower than that of most cancers, such as cervical cancer (52%), rectal cancer (51%), and anal cancer (56%) [1]. Approximately 80% to 85% of lung cancer cases are non-small cell lung cancer (NSCLC). Most NSCLCs are resistant to chemotherapy therapy [2]. Hence, designing more effective chemotherapeutic drugs is necessary for NSCLC treatment in patients, especially for metastatic patients.

Epithelial-mesenchymal transition (EMT) is an important step in cancer development*,* supporting metastasis, and provides novel treatment strategies for cancer therapy. Nevertheless, EMT also contributes to chemoresistance in NSCLC [3]. After activation of the EMT process, epithelial cells changed their phenotype to become mesenchymal stem cells by losing their cell polarity and cell-cell adhesion while gaining migratory, invasive, and cancer stem cell-like properties [4]. Transforming growth factor beta (TGF-β) is a multifunctional cytokine that promotes tumor invasion and migration by inducing EMT in NSCLC cells. Therefore, blocking TGF-β-induced EMT signaling may provide a new therapeutic strategy for NSCLC cancer therapy.

In mammals, there are three major isoforms of TGF*-*β, namely TGF*-*β1*,* -β2 and -β3 [5]. Among them, TGF-β1 is the most abundantly and most widely studied in NSCLC [6]. TGF-β1 ligands bind to TGF-β receptor TGF-βRII, which in turn activates TGF-βRI. Then, the activated TGFβRI further directly phosphorylates SMAD2 and SMAD3. Located mostly in the cytoplasm, these phosphorylated SMAD2 and SMAD3 form a trimeric complex with SMAD4 and translocate into the nucleus. After translocation, the trimeric complex interacts with transcription factors such as Snail and Zeb1 to suppress epithelial marker genes, such as E-cadherin, and induce mesenchymal marker genes, such as vimentin. Thus, the SMAD signaling pathway is activated as an important step in the progression of TGF-β1-induced EMT, making it an attractive therapeutic target for preventing TGF-β1-induced EMT [7].

Resveratrol is a natural phytoalexin compound (stilbenoid) naturally found in plants and exists abundantly in red wine [8]. It has attracted interest from many researchers due to its multiple health benefits, including cardiovascular protection, as well as anti-inflammatory, anti-metastatic and anti-cancer activities. It has been reported that resveratrol suppressed TGF-β1-induced EMT in the human breast cancer cell line MDA-MB-231 [9], human colorectal cancer cell lines LoVo, HCT116 [10], and SW480 [11], and the human lung cancer cell line A549 [12]. Resveratrol can be oligomerized by the oxidative coupling of two to eight units of resveratrol to form oligostilbenoids in diverse plant families [13]. ε-Viniferin is a trans-resveratrol dehydrodimer, α-viniferin is a resveratrol trimer, and kobophenol A is a resveratrol tetramer. [14]. However, the anti-EMT effects of α-viniferin, ε-viniferin, and kobophenol A have not yet been well investigated. The aim of the present study is to evaluate the effect of α-viniferin, ε-viniferin, and kobophenol A on TGF-β1-induced EMT in lung, breast and bone cancer cell lines. To the best of my knowledge, this is the first study examining the anti-EMT effects of α-viniferin, ε-viniferin, and kobophenol A on cancer cells in vitro and in vivo.

## 2. Materials and Methods

### 2.1. Chemicals

Resveratrol (CAS Number 501-36-0, Purity: ≥99%), ε-viniferin (CAS Number 62218-08-0, Purity: ≥99.5%), SB 431542 (CAS Number 301836-41-9, Purity: ≥98%), and [3-(4, 5-dimethylthiazol-2-yl) -2,5-diphenyl tetrazolium bromide] (MTT) (CAS Number: 298-93-1) were purchased from Sigma-Aldrich, Burlington, MA, USA. α-Viniferin (CAS Number 62218-13-7, Purity: ≥98%) and kobophenol A were purchased from SunHank Technology, Tainan City, Taiwan. N-Acetyl-L-cysteine (CAS Number 616-91-1) was purchased from Merck Millipore, Burlington, MA, USA. Reactive oxygen species (ROS) Detection Assay Kits (Catalog No. K936-100) were purchased from BioVision. Anti-β-actin antibody (Cat No. GTX109639), anti-vimentin, anti-MMP2, anti-Slug, anti-Zeb1, anti-Snail, anti-E-cadherin, anti-p-SMAD2, anti-p-SMAD3 and ATP-binding cassette sub-family G member 2 (ABCG2) antibody were purchased from GeneTex.

### 2.2. Cell Culture

A549, HOS, U2OS, NCI-H460 and NCI-H450 cell lines were obtained from Bioresource Collection and Research Center (BCRC, Hsinchu, Taiwan). Cells were cultured and maintained in Dulbecco’s Modified Eagle’s Medium (DMEM) supplemented with 10% fetal bovine serum (FBS) at 37 °C in 5% CO_2_ humidified air.

### 2.3. Cell Treatment for TGF-β1-Induced EMT Analysis

Cells were cultured in DMEM containing 10% FBS during 24 h. After that, the medium was changed, and cells were then incubated in DMEM containing 0.5% FBS for 48–50 h with or without exogenous 10–20 ng/mL TGF-β1 or 10–20 ng/mL IL-1β. To block TGF-β1-induced EMT, cells were pre-incubated with resveratrol, α-viniferin, ε-viniferin, kobophenol A, or SB 431542 for 1 h.

### 2.4. Cell Invasion Assay

Before invasion assays, the 8.0 μm polycarbonate basement membrane (Corning^®^ Transwell^®^ polycarbonate membrane cell culture inserts) was reconstituted with matrigel (30 mg/well, Corning^®^ Matrigel^®^ Matrix). For invasion assays, A549 cells were seeded in the upper side of the polycarbonate membrane in DMEM containing 0.5% FBS with or without α-viniferin or ε-viniferin induced by 10 ng/mL TGF-β1 or 10 ng/mL IL-1β. Conditioned media from *NIH3T3* cells was placed in the lower chambers as a chemoattractant. After incubation for 48 h, invasive cells in the lower chamber were fixed in formaldehyde and stained with crystal violet.

### 2.5. In Vitro Wound-Healing Assay

A549 cells were plated in 6-well plates for 24 h. After that, a straight scratch was gently made with a 200 mL yellow pipette tip in the center of each well. A549 cells were pre-incubated with or without resveratrol, α-viniferin, ε-viniferin, and kobophenol A for 1 h followed by the addition of 10 ng/mL TGF-β1 for 50 h. Then, the image of the wound area was captured by microscopy, and its size was calculated using the Image J software.

### 2.6. Immunofluorescence Staining Assay

The assay was detected using anti-vimentin, anti-Snail, or anti-E-cadherin primary antibodies. A549, HOS, U2OS, NCI-H460 and NCI-H450 cells were pre-incubated with or without resveratrol, α-viniferin, ε-viniferin, kobophenol A for 1 h followed by the addition of 10–20 ng/mL TGF-β1 or 10–20 ng/mL IL-1β for the indicated time. After 48 h, A549, HOS, U2OS, NCI-H460 and NCI-H450 cells were collected, fixed with formaldehyde and then permeabilized with Triton X-100 in phosphate buffered saline (PBS). After fixation, anti-vimentin, anti-Snail, or anti-E-cadherin antibodies were added to cells for 24 h at 4 °C, followed by the addition of secondary antibodies conjugated to fluorescent dyes for 1 h. Finally, the cells were extensively washed with PBS and mounted onto a glass slide. The images were captured under confocal laser scanning microscopy. The mean fluorescence intensity representing the protein level was analyzed using the Image J software.

### 2.7. Gelatin Zymography Analysis

Cell culture medium was obtained from cultured cells in DMEM containing 0.5% FBS with or without α-viniferin or ε-viniferin induced by 10 ng/mL TGF-β1 for 48 h in 10-cm culture dishes. Fifty-fold-concentrated culture media were mixed with Laemmeli’s sample buffer in the absence of β-mercaptoethanol and separated on sodium dodecyl sulfate polyacrylamide gel electrophoresis (SDS-PAGE) containing 0.1% (*w*/*v*) gelatin. After electrophoresis, the gel was washed in renaturing buffer (2.5% *v*/*v* Triton X-100) to remove SDS and was subsequently transferred to a substrate buffer (50 mM Tris–HCl/5 mM CaCl2/0.02% NaN3, pH 8.0) overnight at 37 °C. The gels were agitated in a staining solution containing 0.25% (*w*/*v*) Coomassie blue in 10% (*v*/*v*) acetic acid and 45% (*v*/*v*) methanol at room temperature for 1 h. Clear bands were visualized after several replacements of a destain solution containing 10% (*v*/*v*) acetic acid and 5% (*v*/*v*) methanol.

### 2.8. Western Blot Analysis

Cells were treated with various agents, as indicated in the figure legends. After treatment, cells were lysed in lysis buffer and centrifuged at 12,000 rpm for 30 min at 4 °C for protein extraction. The protein concentration of each lysate was determined using the Bio-Rad protein assay kit (Bio-Rad Laboratories, Hercules, CA, USA). Each lane was loaded with 50 ug of protein separated on SDS-PAGE and transferred to the polyvinylidene fluoride membranes. Then, membranes were incubated with different primary antibodies, and then with secondary antibodies conjugated with horseradish peroxidase. The immunoreactive bands were visualized by chemiluminescence reagents.

### 2.9. ROS Detection Assay

The assessment of the generated ROS was performed according to the manufacturer’s protocol for ROS Detection Assay Kits (BioVision, Milpitas, CA, USA, Catalog No. K936-100) using a fluorescence microscope.

### 2.10. In Vivo Xenograft Metastasis Experiments

Male BALB/c nude mice (five weeks old) were purchased from the National Laboratory Animal Breeding and Research Center (Taipei, Taiwan). The experiments were approved by the Institutional Animal Care and Use Committee of National Tsinghua University. Mice were injected with A549 cells treated with TGF-β1 via the tail vein (1 × 10^6^ cells in PBS). After 28 days, mice were treated with PBS or 5 mg/kg ε-viniferin five times per week by intraperitoneal administration. Mice were sacrificed at four weeks. The area of the tumor nodule, vimentin expression and histopathological changes in the lung tissue were further evaluated using hematoxylin and eosin (H&E) staining and immunohistochemistry (IHC) staining.

### 2.11. Statistical Analysis

All data were expressed as mean ± standard deviation (SD). The Student’s *t*-test was used to compare the difference between two groups (* *p* < 0.05, ** *p* < 0.01, *** *p* < 0.001).

## 3. Results

### 3.1. α-Viniferin and ε-Viniferin Blocked TGF-β1-Induced Invasion and Migration in NSCLC Cell Line A549

**Figure 1:**
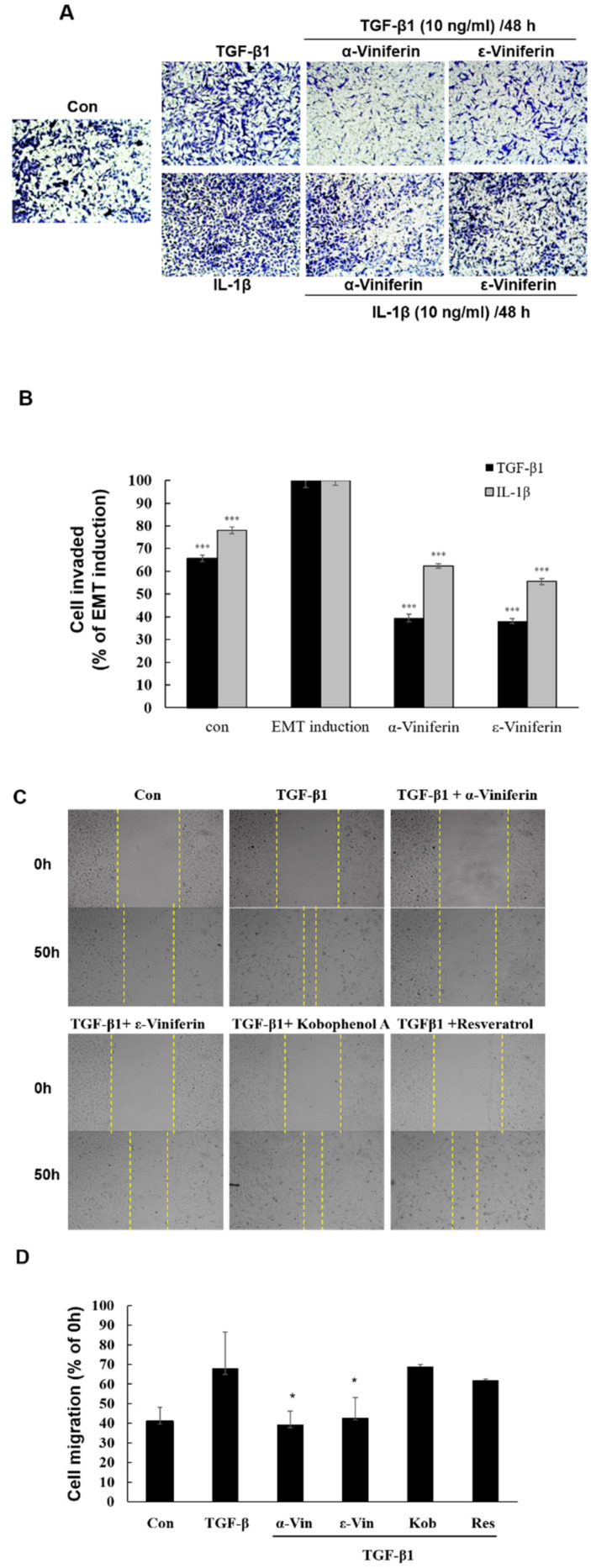
α-Viniferin and ε-viniferin inhibited both the invasion and migration of TGF β1- or IL-1β induced A549 cells. Cells were preincubated with or without 10 μM resveratrol, 10 μM α-viniferin, 10 μM ε-viniferin, or 10 μM kobophenol A for 1 h, and were then treated with TGF β1 (10 ng/mL) or IL-1β (10 ng/mL) for 48–50 h. The invasion and migration abilities of TGF β1- or IL-1β induced A549 cells following treatment with the above compounds were determined using (**A**) wound healing and (**C**) transwell assay. (**B**) Quantification of transwell assay using Image J and represented as the percentage of EMT induction (TGF β1 or IL-1β treatment alone). (**D**) Quantification of wound healinga ssay using Image J and represented as the percentage of 0 h treatment. (* *p* < 0.05, *** *p* < 0.001).

To determine the role of α-viniferin and ε-viniferin in cellular invasion and migration in TGF-β1- or IL-1β-induced A549 cells, this study used a transwell invasion and wound healing assay. α-Viniferin and ε-viniferin significantly suppressed the TGF-β1- or IL-1β-induced invasion or migration of A549 cells (Figure 1). Furthermore, this research aimed to evaluate the effects of α-viniferin and ε-viniferin on the expression of EMT-related markers.

### 3.2. α-Viniferin and ε-Viniferin Inhibited TGF-β1- or IL-1β-Induced Vimentin Expression in NSCLC Cell Lines A549 and NCI-H460

**Figure 2:**
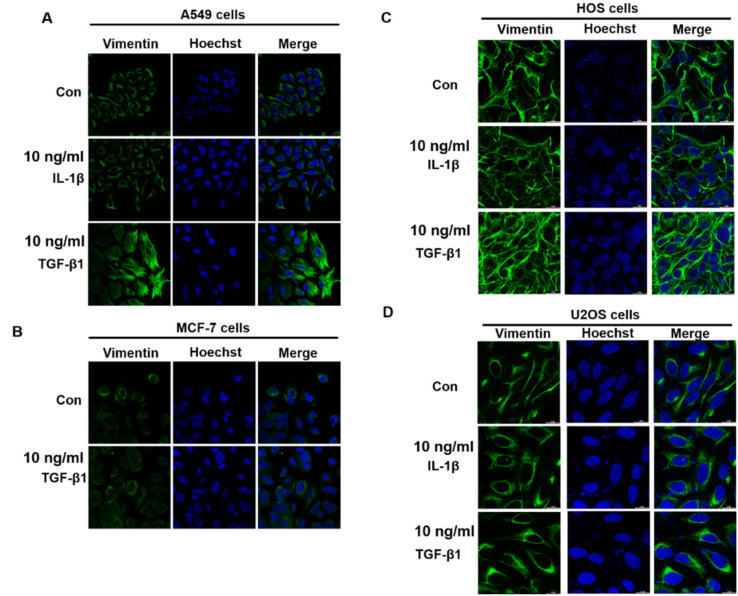

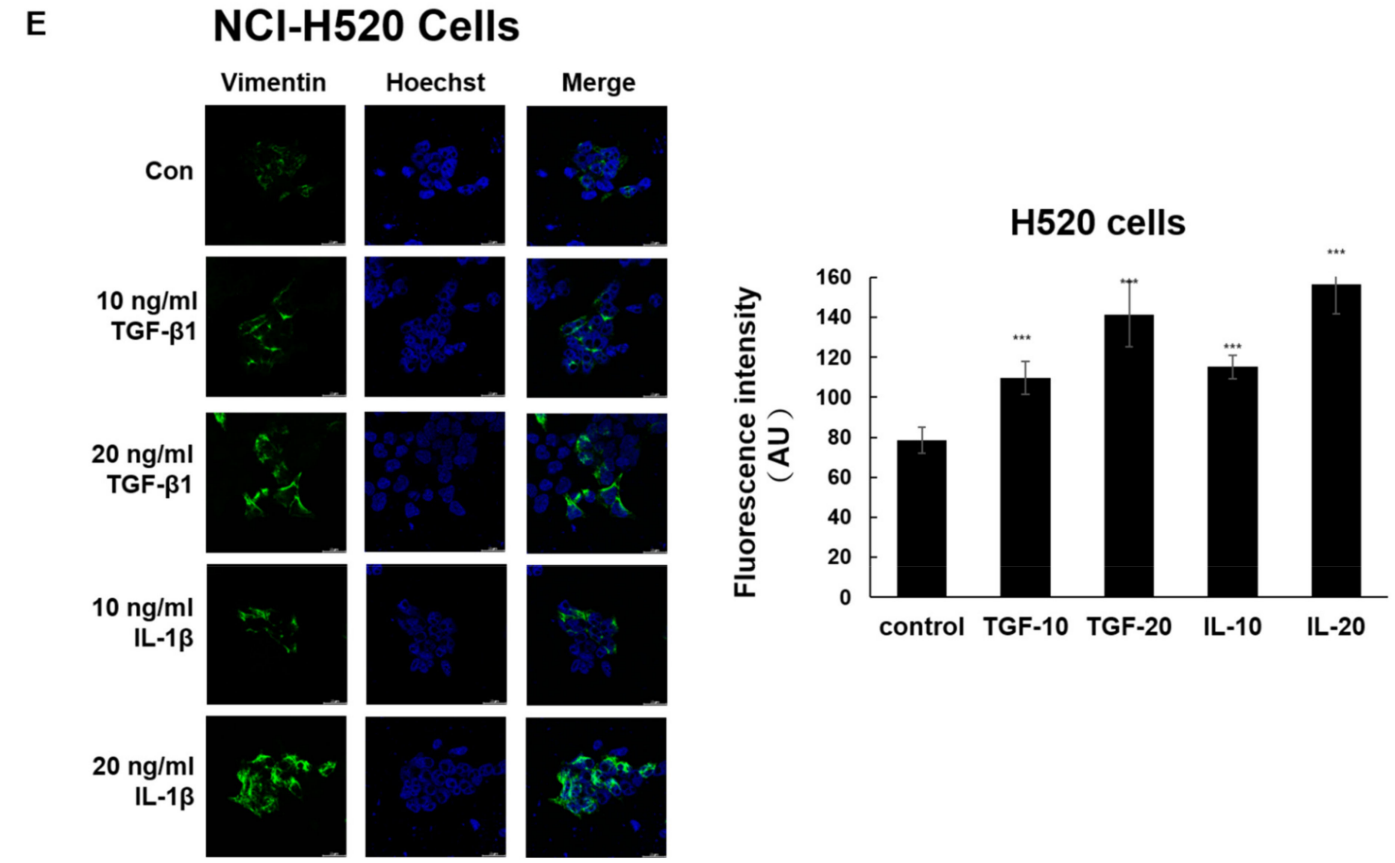
Vimentin was detected by immunofluorescence analysis in TGF β1- or IL-1β-induced (**A**) A549, (**B**) MCF-7, (**C**) HOS, (**D**) U2OS and (**E**) NCI-H520 cells. Cells were treated with or without TGF β1 (10–20 ng/mL) or IL-1β (10–20 ng/mL) for 48 h. The expression of vimentin was detected using primary anti-vimentin antibody. Images show nuclei stained by Hoechst 33,258 staining and viewed under confocal laser scanning microscopy. Quantitative analysis of vimentin fluorescence intensity using Image J. An asterisk (*) indicates a significant difference compared with the control treatment (*** *p* < 0.001). The scale bar represents 20 μm in the images.

**Figure 3:**
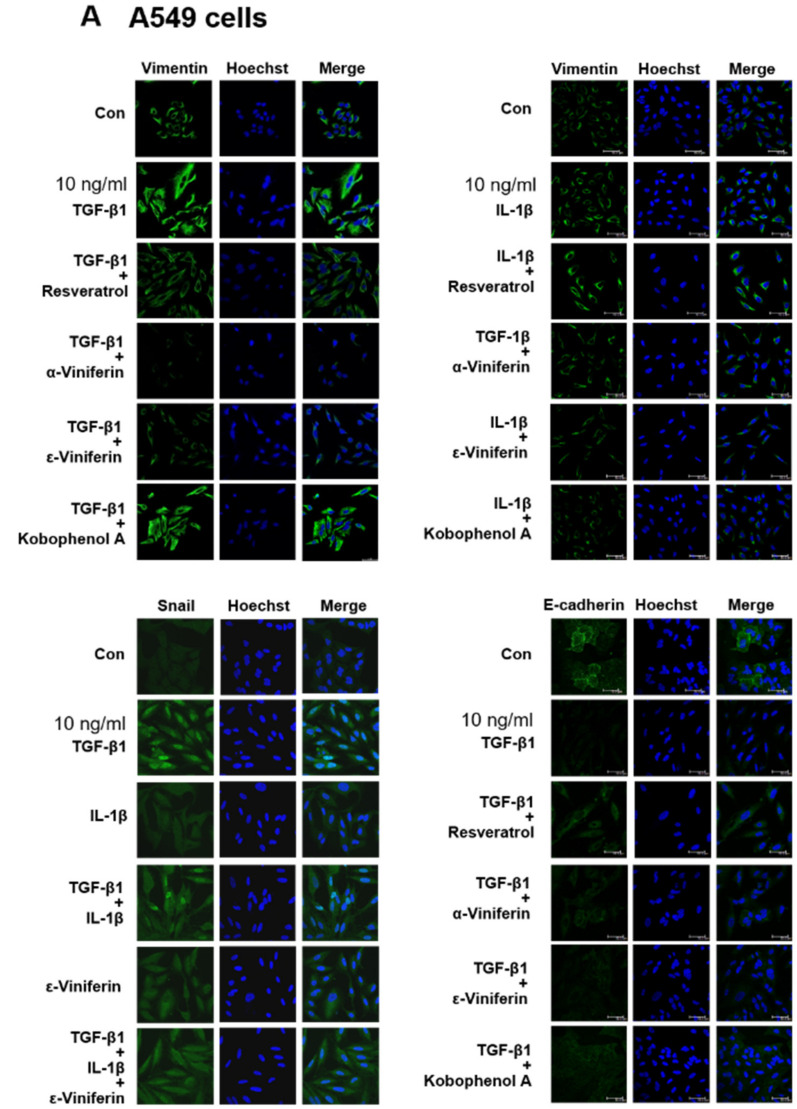

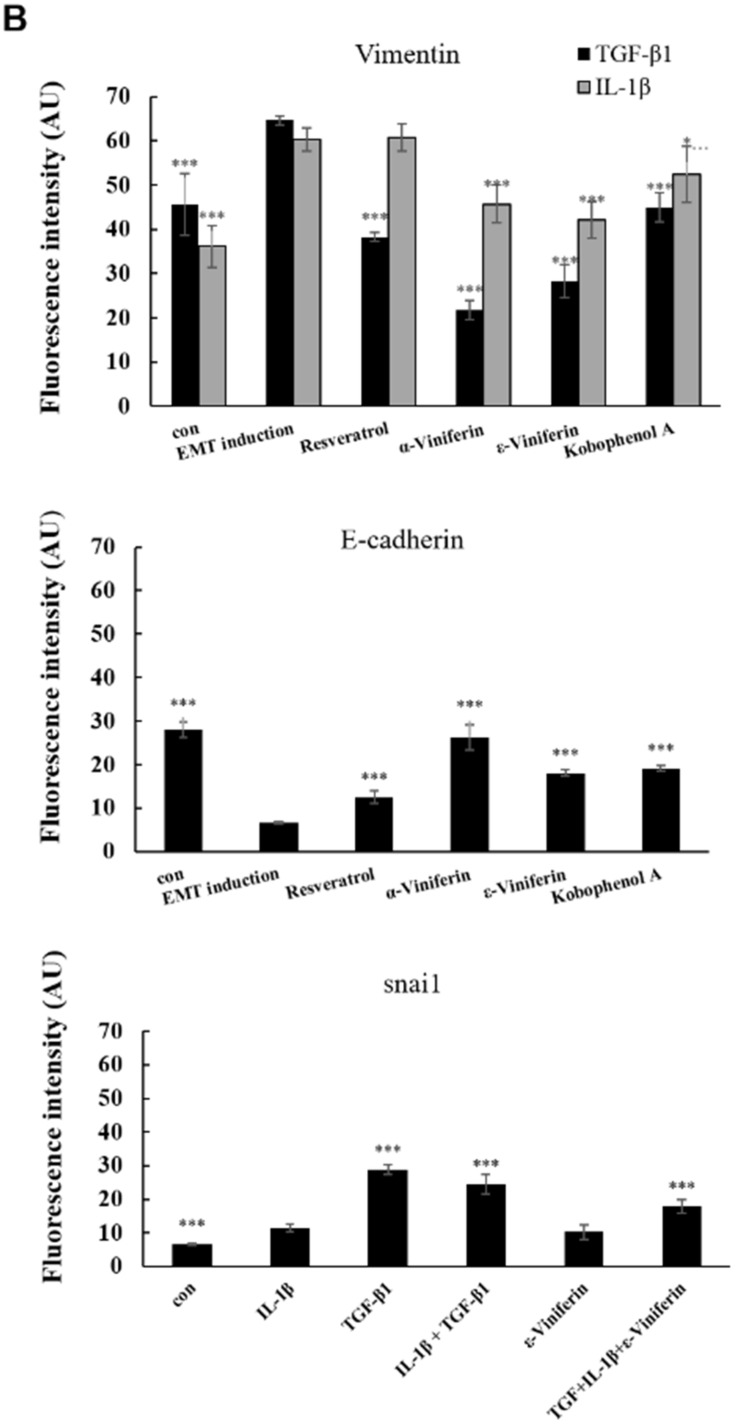

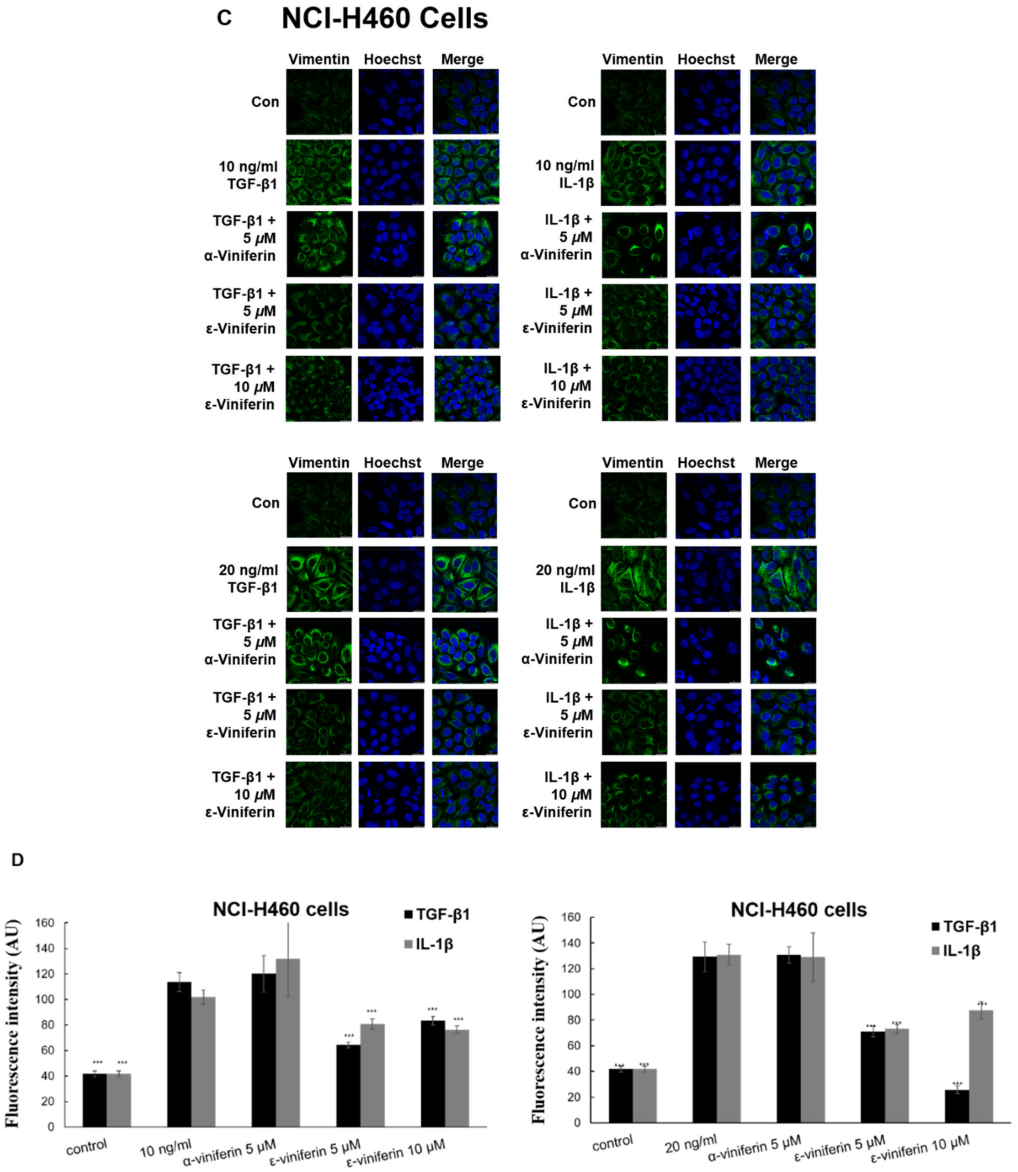
Effects of resveratrol, α-viniferin, ε-viniferin and kobophenol A on vimentin, Snail, and E-cadherin expression in TGF β1- or IL-1β-induced (**A**) A549 and (**C**) NCI-H460 cells. Cells were preincubated with or without 10 μM resveratrol, 5–10 μM α-viniferin, 5–10 μM ε-viniferin, or 10 μM kobophenol A for 1 h, and then treated with TGF β1 (10–20 ng/mL) or IL-1β (10–20 ng/mL) for 48 h. Vimentin, Snail, and E-cadherin expression were detected using anti-vimentin, anti-E-cadherin, and anti-Snail primary antibodies. Images show nuclei stained by Hoechst 33,258 staining and viewed under confocal laser scanning microscopy. (**B**,**D**) Quantitative analysis of vimentin, Snail, and E-cadherin fluorescence intensity using Image J. An asterisk (*) indicates a significant difference compared with the TGF β1 or IL-1β treatment (* *p* < 0.05, *** *p* < 0.001). The scale bar represents 20 μm in the images.

This study takes vimentin expression as an indicator for evaluating the anti-EMT potential of resveratrol, α-viniferin, ε-viniferin, and kobophenol A in TGF-β1- or IL-1β-induced A549, MCF-7, HOS, U2OS, NCI-H460 and NCI-H520 cancer cells using western blotting and immunofluorescence. The immunofluorescence staining of vimentin revealed that TGF-β1 induced vimentin expression in A549, NCI-H460 and NCI-H520 cells, but not in MCF-7, HOS, and U2OS cells (Figure 2). IL-1β (10 ng/mL) only induced vimentin expression in NCI-H520 (Figure 2E), A549 (Figure 3A), and NCI-H460 cells (Figure 3D), but not in MCF-7, HOS, and U2OS cells. Furthermore, the changes in vimentin expression induced by TGF-β1 were significantly decreased by treatment with resveratrol, α-viniferin, ε-viniferin, and kobophenol A in A549 cells (Figure 3A,B). α-viniferin, ε-viniferin, and kobophenol A also inhibited IL-1β-induced vimentin expression in A549 cells. Additionally, 5 μM ε-viniferin also inhibited TGF-β1- or IL-1β- induced vimentin expression in NCI-H460 cells, but α-viniferin did not (Figure 3C,D).

### 3.3. α-Viniferin, ε-Viniferin, and SB431542 Blocked TGF-β1-Induced Vimentin, Zeb1, Snail, MMP2, ABCG2, and SMAD2/SMAD3 Activation in NSCLC Cell Line A549

**Figure 4:**
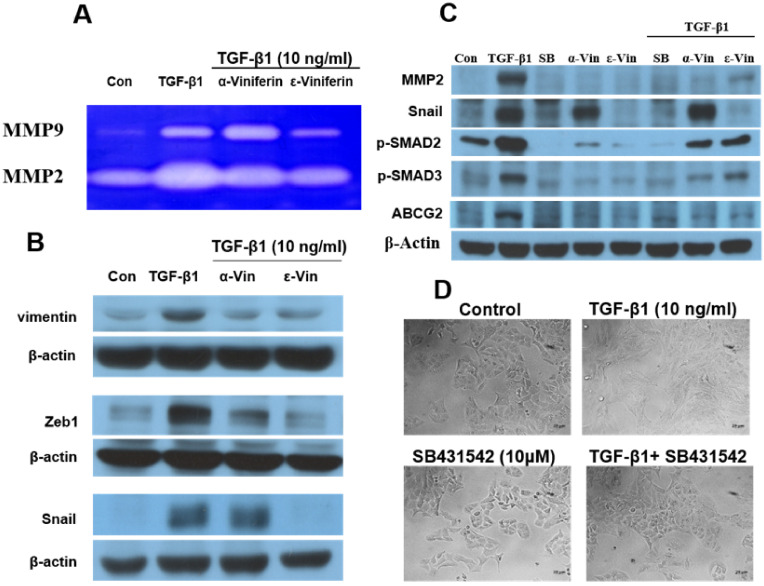
Effects of α-viniferin, ε-viniferin, and SB431542 on EMT-related protein expression in TGF-β1-induced A549 cells. Cells were preincubated with or without 10 μM α-viniferin, 10 μM ε-viniferin, or SB431542 for 1 h, and then stimulated with TGF β1 (10 ng/mL) for 48 h. (**A**) MMP-2 and MMP-9 activities were determined by gelatin zymography. (**B**,**C**) Expression levels of MMP2, vimentin, Zeb1, Snail, p-SMAD2, p-SMAD3, and ABCG2 were detected using western blotting with the primary antibody. β-Actin served as a loading control. (**D**) Morphological changes of A549 cells treated with TGF-β1 and TGF-β1 plus SB431542 compared with the control.

The results of the gelatin zymography analysis revealed that ε-viniferin blocked TGF-β1*-*induced MMP2 and MMP9 activity but that α-viniferin only decreased TGF-β1-induced MMP2 activity (Figure 4A). Additionally, this study evidenced TGF*-*β1-induced MMP2, vimentin, Zeb1, Snail*,* p*-*SMAD2, p-SMAD3, and ABCG2 expression in A549 cells. α-viniferin and ε-viniferin pretreatment could block TGF-β1-induced MMP2*,* vimentin, Zeb1, p*-*SMAD2, p-SMAD3, and ABCG2 expression. ε-viniferin also decreased Snail expression in TGF-β1-induced *A*549 cells. SB431542 (TGF*-*β1 receptor inhibitor), which showed similar effects as ε-viniferin, suppressed the TGF-β1-induced MMP-2, Snail, ABCG2, P-SMAD2, and P-SMAD3 expression and restored the morphological changes in A549 cells (Figure 4C,D). These results suggested that α-viniferin, ε-viniferin, and SB431542 repressed TGF*-*β1-induced EMT in A549 cells, which is probably associated with blocking TGF*-*β1/SMAD2/3-dependent signaling pathways.

### 3.4. Induction of ROS Production in A549 and NCI-H460 Cells by TGF-β1 and Protective Effect of α-Viniferin and ε-Viniferin against TGF-β1-Induced ROS Production

**Figure 5:**
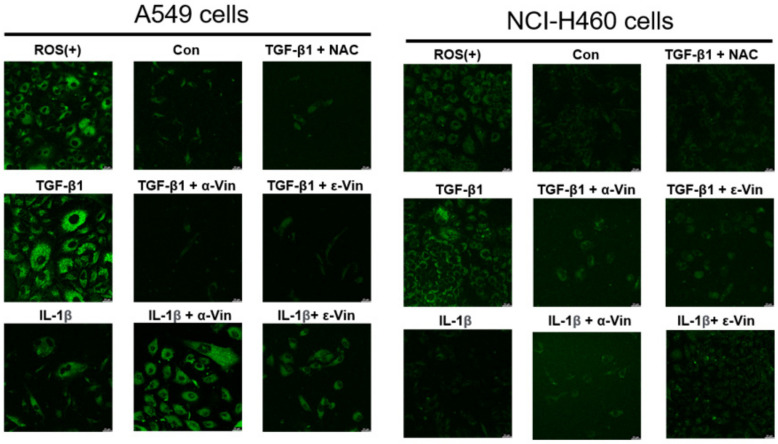
Effects of α-viniferin and ε-viniferin on ROS levels in TGF-β1-induced A549 and NCI-H460 cells. ROS generation in A549 and NCI-H406 cells following various treatments was measured using ROS Detection Assay Kits. The scale bar represents 20 μm in the images.

Yazaki et al. reported that the ROS mediated TGF-β1-induced EMT in A549 cells [15]. To investigate whether TGF-β1 stimulated ROS generation in A549 and NCI*-*H460 cells, this study used ROS detection assay kits to detect intracellular ROS levels. The ROS inducer and TGF-β1 significantly increased ROS production in A549 and NCI*-*H460 cells, but α-viniferin, ε-viniferin, or N-acetyl-L-cysteine (NAC) co-treatment completely abrogated TGF-β1-induced ROS production in A549 and H460 cells (Figure 5).

### 3.5. ε-Viniferin Inhibited A549 Cell Metastasis In Vivo

**Figure 6:**
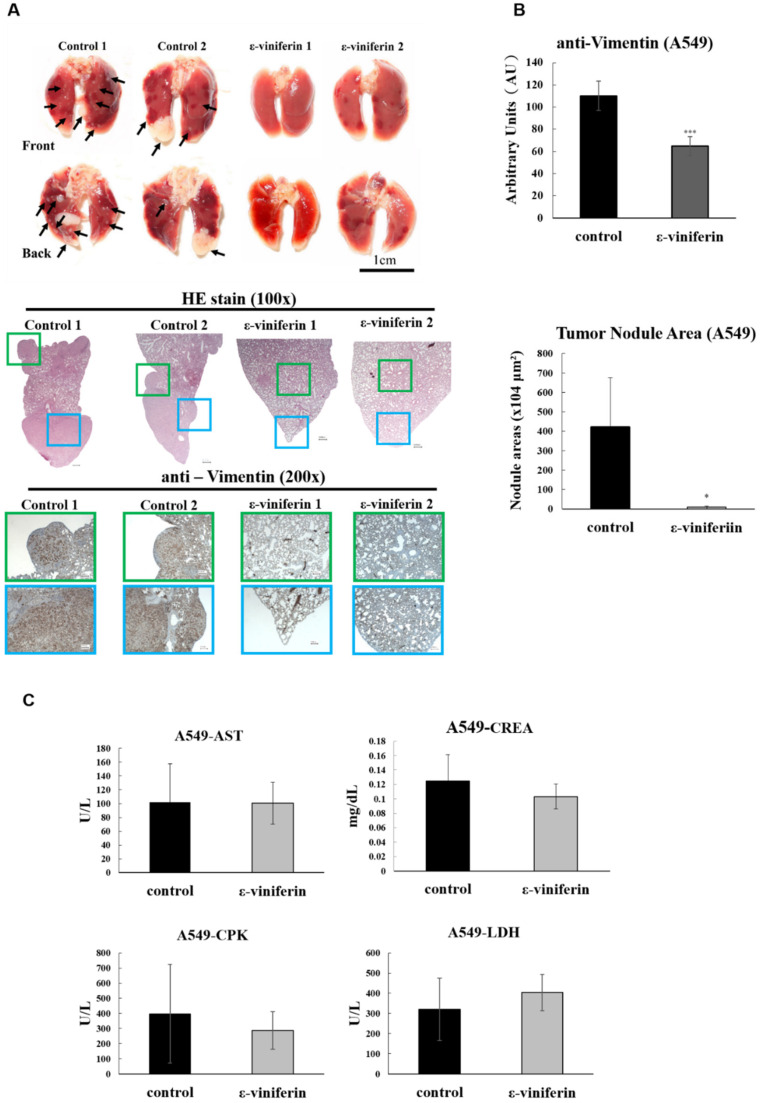
ε-viniferin inhibited the metastatic ability of A549 cells in vivo. A lung metastasis mouse model was generated as described in Materials and Methods. Nude mice were injected with A549 cells treated with TGF-β1 via the tail vein and were intraperitoneally administered 5 mg/kg ε-viniferin five times per week for four weeks. (**A**) Representative images are shown for the lung with metastatic nodules (black arrows). The tumor morphology and vimentin expression in lung tissue were examined using H&E and IHC staining (original magnification ×100–200). Green and blue squares indicated the areas magnified. (**B**) Quantitative analysis of vimentin and tumor nodule area using Image J. The scale bar represents 10 μm in the images. (**C**) The AST, LDH, CPK, and creatinine levels in serum were determined. All values were expressed as mean ± SD. An asterisk (*) indicates values significantly different from the control (* *p* < 0.05; *** *p* < 0.001).

To confirm the anti-metastatic activity of ε-viniferin in vivo, this study established a metastatic model in nude mice by injecting cells into the tail veins of mice. Representative images of lung tissue are shown in Figure 6. The histological analysis of the lung tissue revealed a significantly decreased metastatic nodule area in A549*-*xenografted nude mice treated with ε-viniferin compared with control mice. Vimentin expression was also detected by the immunohistochemical analysis of lung tumors treated with ε-viniferin. Vimentin, as a marker of TGF-β1-induced-EMT, was suppressed by ε-viniferin, but not by the control treatment. The serum values of AST, LDH, CPK, and creatinine were detected to evaluate side effects caused by ε-viniferin. As shown, respectively, in Figure 6C, α-viniferin and ε-viniferin did not influence the liver or kidney function in mice. These results further supported the anti-metastatic activities of ε-viniferin in vivo.

## 4. Discussion

Despite advances in NSCLC diagnosis and treatment, the treatment of NSCLC patients with distant metastases remains a great challenge [16,17]. Resveratrol, having few side effects, has been widely studied and has been known to possess anti-EMT and anti-metastasis properties [10,12], but research on oligomeric resveratrol is still scarce. Previous findings have shown that ε-viniferin had a higher concentration in grapevine leaves and exhibited higher biological activities than resveratrol [18]. The poor bioavailability of resveratrol is mainly due to its poor absorption. Unlike resveratrol, α-viniferin and ε-viniferin could be absorbed extremely quickly in vivo [19,20]. Because the bioavailabilities of α-viniferin and ε-viniferin were significantly higher than that of resveratrol, they could be suitable for the development of bioactive therapeutic compounds.

TGF-β1-induced EMT had also been implicated in chemoresistance, specifically of platinum therapies in ovarian cancer and tamoxifen in breast cancer [21]. ABCG2, as a marker for cancer stem cells, plays a major role in multidrug resistance in NSCLC. ABCG2 also contributes to EMT and cancer metastasis, and is a direct target of TGF-β1 in tumor cells [22]. TGF-β1 has been reported to upregulate ABCG2 expression via the SMAD-dependent signaling pathway in various human cancers, including breast and pancreatic cancers [23,24]. However, it is not currently known whether TGF-β1 could directly regulate the expression of ABCG2 in lung cancer. The present findings showed that TGF-β1 could induce ABCG2 protein expression in A549 cells and that the increase was reversed by α-viniferin, ε-viniferin, and SB431542 (Figure 4).

In addition to TGF-β1, IL-1β has been demonstrated to induce EMT in normal kidney (HK-2 cells) [25], stomach cancer (SNU719) [26], and breast cancer (MCF-7) [27]. However, the results were not consistent in human lung epithelial cell lines. Borthwick et al. reported that IL-1β alone did not induce EMT in primary bronchial epithelial cells (PBECs) derived from normal lung tissue [28]. Li et al. reported that IL-1β alone could induce EMT in the A549 lung cancer cell line [29]. Other studies found that IL-1β augmented the TGF-β1-induced EMT of A549 cells [30,31]. The present findings confirmed previous results according to which IL-1β alone could induce EMT via increasing vimentin expression in A549, NCI-H460 and NCI-H520 cells (Figure 2 and Figure 3). However, the question of whether IL-1β augments TGF-β1-induced EMT in A549, NCI-H460 and NCI-H520 cells needs to be investigated.

Vimentin overexpression in different cancer cell lines and tissues was shown to be an independent positive prognostic factor for poor survival in patients with resected NSCLC [32]. As a canonical marker of EMT, vimentin found in the cytoplasm of mesenchymal cells was involved in maintaining cytoarchitectural integrity [33]. Previous studies found an upregulated expression of vimentin in metastatic lung tissues compared with normal lung tissues, also suggesting a possible association of vimentin overexpression with metastatic lung tumors. Therefore*,* vimentin overexpression can be a potential marker for predicting metastasis and can serve as an attractive potential therapeutic strategy for invasive lung cancer. The present findings indicated that ε-viniferin could inhibit TGF-β1-induced EMT in vitro (Figure 3 and Figure 4) and in vivo (Figure 6) by targeting vimentin expression. Glycosylated vimentin is downregulated in metastatic lung cancer and is suggested to represent novel biomarkers for diagnosis [34]. Thus, the glycosylated vimentin expression may represent an avenue for new treatment strategies for patients with metastatic lung cancer. Furthermore, the effect of α-viniferin and ε-viniferin on glycosylated vimentin expression needs to be investigated.

In conclusion, this study provided the first evidence showing that ε-viniferin significantly inhibited lung metastasis in A549 cell xenograft metastatic mouse models. Both α-viniferin and ε-viniferin decreased MMP-2 protein expression and enzyme activity in TGF*-*β1-induced A549 cells. α-viniferin and ε-viniferin also reversed the expression of resistance*-*associated protein (ABCG2), EMT*-*related protein (vimentin and Zeb1), and ROS generation in TGF*-*β1-induced A549 cells through the downregulation of the P*-*SMAD2/P-SMAD3-dependent signaling pathway responsible for key EMT. Targeting the TGF-β1/SMAD signaling pathway as an anti-cancer treatment may be useful in the prevention of drug resistance, EMT and cancer metastasis.

## Data Availability

Not applicable.
